# proGenomes3: approaching one million accurately and consistently annotated high-quality prokaryotic genomes

**DOI:** 10.1093/nar/gkac1078

**Published:** 2022-11-21

**Authors:** Anthony Fullam, Ivica Letunic, Thomas S B Schmidt, Quinten R Ducarmon, Nicolai Karcher, Supriya Khedkar, Michael Kuhn, Martin Larralde, Oleksandr M Maistrenko, Lukas Malfertheiner, Alessio Milanese, Joao Frederico Matias Rodrigues, Claudia Sanchis-López, Christian Schudoma, Damian Szklarczyk, Shinichi Sunagawa, Georg Zeller, Jaime Huerta-Cepas, Christian von Mering, Peer Bork, Daniel R Mende

**Affiliations:** Structural and Computational Biology Unit, European Molecular Biology Laboratory, 69117 Heidelberg, Germany; Biobyte solutions GmbH, Bothestr. 142, 69117 Heidelberg, Germany; Structural and Computational Biology Unit, European Molecular Biology Laboratory, 69117 Heidelberg, Germany; Structural and Computational Biology Unit, European Molecular Biology Laboratory, 69117 Heidelberg, Germany; Structural and Computational Biology Unit, European Molecular Biology Laboratory, 69117 Heidelberg, Germany; Structural and Computational Biology Unit, European Molecular Biology Laboratory, 69117 Heidelberg, Germany; Structural and Computational Biology Unit, European Molecular Biology Laboratory, 69117 Heidelberg, Germany; Structural and Computational Biology Unit, European Molecular Biology Laboratory, 69117 Heidelberg, Germany; Royal Netherlands Institute for Sea Research (NIOZ), Department of Marine Microbiology & Biogeochemistry, 1797 SZ, ’t Horntje (Texel), Netherlands; Department of Molecular Life Sciences and Swiss Institute of Bioinformatics, University of Zurich, 8057 Zurich, Switzerland; Institute of Microbiology, Department of Biology and Swiss Institute of Bioinformatics, ETH Zurich, Vladimir-Prelog-Weg 4, 8093 Zurich, Switzerland; Department of Molecular Life Sciences and Swiss Institute of Bioinformatics, University of Zurich, 8057 Zurich, Switzerland; Centro de Biotecnología y Genómica de Plantas, Universidad Politécnica de Madrid (UPM) - Instituto Nacional de Investigación y Tecnología Agraria y Alimentaria (INIA-CSIC), Campus de Montegancedo-UPM, 28223, Pozuelo de Alarcón, Madrid, Spain; Structural and Computational Biology Unit, European Molecular Biology Laboratory, 69117 Heidelberg, Germany; Department of Molecular Life Sciences and Swiss Institute of Bioinformatics, University of Zurich, 8057 Zurich, Switzerland; Institute of Microbiology, Department of Biology and Swiss Institute of Bioinformatics, ETH Zurich, Vladimir-Prelog-Weg 4, 8093 Zurich, Switzerland; Structural and Computational Biology Unit, European Molecular Biology Laboratory, 69117 Heidelberg, Germany; Centro de Biotecnología y Genómica de Plantas, Universidad Politécnica de Madrid (UPM) - Instituto Nacional de Investigación y Tecnología Agraria y Alimentaria (INIA-CSIC), Campus de Montegancedo-UPM, 28223, Pozuelo de Alarcón, Madrid, Spain; Department of Molecular Life Sciences and Swiss Institute of Bioinformatics, University of Zurich, 8057 Zurich, Switzerland; Structural and Computational Biology Unit, European Molecular Biology Laboratory, 69117 Heidelberg, Germany; Max Delbrück Centre for Molecular Medicine, 13125 Berlin, Germany; Department of Bioinformatics, Biocenter, University of Würzburg, 97074 Würzburg, Germany; Yonsei Frontier Lab (YFL), Yonsei University, 03722 Seoul, South Korea; Department of Medical Microbiology, Amsterdam University Medical Centers, Amsterdam, The Netherlands

## Abstract

The interpretation of genomic, transcriptomic and other microbial ‘omics data is highly dependent on the availability of well-annotated genomes. As the number of publicly available microbial genomes continues to increase exponentially, the need for quality control and consistent annotation is becoming critical. We present proGenomes3, a database of 907 388 high-quality genomes containing 4 billion genes that passed stringent criteria and have been consistently annotated using multiple functional and taxonomic databases including mobile genetic elements and biosynthetic gene clusters. proGenomes3 encompasses 41 171 species-level clusters, defined based on universal single copy marker genes, for which pan-genomes and contextual habitat annotations are provided. The database is available at http://progenomes.embl.de/

## INTRODUCTION

Microbiology and microbiome research have made great advances over the recent decades, in large part thanks to the availability of large-scale genomics data (1). Nowadays, hundreds of thousands of genomes are available and microbiology has become a data-intensive as well as data-driven research field. Sequencing has become available at low costs, fueling the continued exponential increase of sequenced bacterial and archaeal genomes (2,3). This increase in data has led to many new discoveries and a better understanding of the biology of microbes facilitated by comparative genomics e.g. (4,5). To leverage comparative studies of these genomes for scientific discoveries (6), high-quality genomes with consistent annotations are required.

The proGenomes (prokaryotic genomes) database provides researchers with such high-quality genomes in a framework that can serve multiple biological disciplines ranging from evolution and ecology to medicine. By further providing easy access and many different annotation layers at once, proGenomes enables researchers of all levels of expertise in genomics to perform comparative analyses and gain scientific insight. Other prominent examples of genomics databases are the NCBI RefSeq database (7), which enables public access to a comprehensive set of genomes but only provides minimal annotations, Ensembl Bacteria (8), the DOE’s Joint Genome Institute Integrated Microbial Genomes & Microbiomes (JGI IMG/M) database (9), the PATRIC (Pathosystems Resource Integration Center) database (10) of the Genome Taxonomy Database (GTDB) (11,12), which focuses on a consistent taxonomy across the bacterial and archaeal tree of life. The latter is a highly important effort, as many other databases suffer from phylogenetic and taxonomic inconsistencies, often due to submitter errors (13–15). However, similar consistency is needed for other types of annotations, such as gene functions, phenotypic data and habitat information. Similarly, habitat annotation is often neglected in existing databases, and indeed for most isolates the habitat is insufficient for ecological analysis due to a lack of a unified ontology. Even if habitats are described, these are often incomparable. Different groups have set out to establish habitat databases and ontologies such as the Microbe Atlas Project (MAP), the Earth Microbiome Project (EMP), ENVO and JGI Gold (16–19). For example, the MAP uses 16S rRNA sequences from studies across the globe to map taxa to habitats (17). proGenomes3 integrates and links to MAP now, further improving the existing habitat annotations.

While general functional annotations are of utmost importance for comparative genomics (and are included in proGenomes via eggNOG annotations (20,21)), some genomic elements require focused and dedicated approaches. For example, mobile genetic elements (MGEs) cover on average 13% of prokaryotic genomes but their annotation still remains poor. Most available databases focus on annotation of a particular MGE type (22–24) and an overview of all MGEs within a genome for comparative analysis is missing. As a new feature within proGenomes3, we identified MGEs for all representative genomes using recombinase marker genes which were further annotated as transposable elements, phages, phage-like elements, conjugative elements, mobility islands, and integrons based on a previously described framework for mobile element annotation (25).

Ensuring a high quality of genomes requires an assessment of genomic completeness and contamination. Recent advances in this area have led to the development of the CheckM and GUNC tools (26–28). proGenomes3 applies these quality control tools to all included genomes and consistently annotates them taxonomically and functionally. These are combined and linked with habitat information, adding further value for comparative analyses and metagenomic studies. The updated version provides ten times as many genome sequences and annotations compared to proGenomes2 and has a higher phylogenetic coverage. Additionally, these genomes are now linked to a number of additional resources enabling direct access to a complete picture of genomes of interest. A number of workflows were improved for proGenomes3, enabling the processing of nearly one million genomes and four billion genes, while increasing the number of annotation tracks. In essence, proGenomes3 provides easy access to everything needed for comparative analyses of prokaryotic genomes. The database is available at http://progenomes.embl.de/

## DATABASE CONSTRUCTION AND CHARACTERISTICS

The proGenomes3 website allows users to access and browse microbial genomes. A search function gives direct access via the NCBI assembly ID or the taxonomic name of the organism, species or clade which can be interactively explored. Subsets of genomes can be downloaded directly.

Future updates will be in regular intervals and major upgrades of the underlying computational pipeline are planned every 2 years. For the current release, proGenomes 3.0, genomes were downloaded on 30 September 2021.

### Genome collection

We downloaded all bacterial and archaeal genomes that were available from the NCBI Nucleotide database on 30 Sep 2021. All genomes were annotated using Prokka (1.14.5). Closed genomes were accepted as high quality. Incomplete genomes were quality filtered using CheckM (1.0.13) and GUNC (1.0.1) (CheckM: completeness > 90% and contamination < 5%; GUNC: contamination < 5% and clade separation score < 0.45) (20,21). After removing 117 723 genomes (15 928 genomes were filtered out due to GUNC and 106 766 due to CheckM, overlap: 8319), this resulted in a total of 907 388 high-quality genomes. High-quality, yet incomplete genomes are suitable for most genomics analysis, but might still miss core genes, hence parameters should be adjusted accordingly when using these genomes for specific follow-up analyses.

### Delineating species using specI clusters

The specI method delineates genomes into accurate and consistent species clusters (29). Generally, these agree with the existing species definitions based on morphological and phenotypic features. We employed a divide-and-conquer strategy to generate specI clusters: First, genomes were subdivided into broader clusters by using single linkage clustering at a 90% Average Nucleotide Identity (ANI) cutoff calculated using Mash (30). Afterwards, specI species clusters were generated for every one of these broader clusters as described for previous proGenomes versions (29). In short, a set of 40 universal, single-copy marker genes (31,32) was extracted from all genomes and pairwise genome-to-genome identities were calculated with vsearch (v1.8.0) (33) as a length-weighted average of the nucleotide identities. Pairwise identities were converted into distances and clustered using average linkage clustering with a distance cutoff of 3.5% (96.5% nucleotide identity). The 907 388 proGenomes3 genomes were delineated into 41 171 specI species clusters. This is >3-fold increase in specI clusters when compared to proGenomes2 (34). Genomes and specI clusters were taxonomically annotated using GTDB (version 202) (12) and the NCBI taxonomy (version from 1 Oct 2021).

### Selection of representative genomes

Due to the availability of multiple genomes for many species and strains, genomic databases have to handle an increasing amount of redundancy. Many applications in genomics require non-redundant genomes (35,36), and accordingly proGenomes provides a non-redundant set of 41 171 representative genomes as well as habitat-specific subsets. These representative genome collections are readily available for direct download from the proGenomes website.

**Figure 1.**
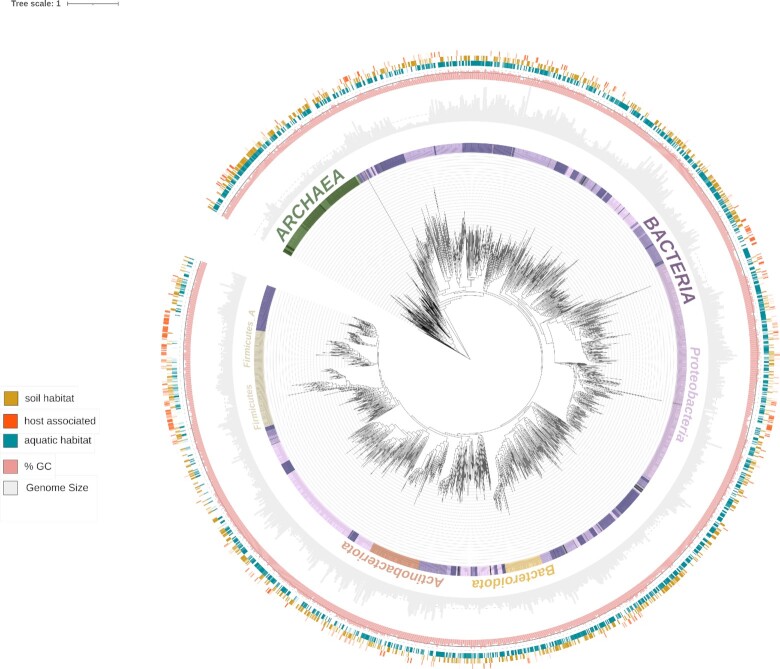
Phylogenetic tree of 41k representative genomes collapsed at the order level (GTDB taxonomy). Phylum, habitat as well as GC content and genome size were displayed as rings surrounding the tree.

We selected one representative genome per specI cluster. Some strains are *de facto* representatives of a species within parts of the scientific community, for example *Mycobacterium tuberculosis* H37Rv. To make sure that these genomes are included in the set of representatives, a whitelist was compiled including genomes from highly important strains and is available on the proGenomes website. However, most clusters do not contain genomes in the whitelist. For these, we first sub-selected all complete genomes and then chose the genome of the most highly cited strain (37). If no complete genomes were part of the specI cluster, the genome with the highest N50 was selected. We provide a phylogenetic tree of all representative genomes to facilitate further analyses (Figure 1). The phylogenetic tree was built from a set of 40 universal, single-copy marker genes (29,32), which were separately aligned with FAMSA v2 (38). The concatenated alignment was used to generate a tree using FastTree/2.1.11-GCC-8.2.0–2.31.1 (39). The tree was annotated and visualized using ete4 (40).

### Pan-genomes

Pan-genomes have been used to understand the genomic variability within species (4). Within proGenomes3, the pan-genome for every specI species cluster is provided as a non-redundant gene set.

These were generated by clustering using mmseqs2 (version 13.45111) (exact parameters used: –min-seq-id 0.95 -c 0.90 –cov-mode 0). Using this process, we reduced the total number of genes from ca. 4 billion to ca. 200 million while providing a more comprehensive coverage of each species’ functional repertoire.

### Functional annotation

Consistent functional annotation of microbial genomes is crucial for comparative analyses and to understand phenotype, lifestyle and ecological role. Providing these annotations is one of the main focal points of proGenomes. Overall annotations were assigned using eggNOG-mapper for eggNOG 5.0 (20) which assigned protein coding sequences to functionally annotated orthologous groups. A total of 3.7 billion protein-coding genes received eggNOG annotations.

To provide Carbohydrate-active Enzyme (CAZy) annotations, we utilized CAZy sequences obtained from dbCAN2 (41) to obtain optimal HMM *P*-values in a cross-validation scheme. Briefly, we divided module sequences of all (sub)families into training and testing sets and computed (sub)family-wise HMM *P*-value cutoffs that yield maximum classification performance using the testing set as positive instances (for a given (sub)family) and all other sequences as negative instances. Using these optimized *P*-values for each family, we then annotated pangenomes using the pyhmmer suite and transferred annotations to ORFs of all individual genomes.

proGenomes3 provides gene-level annotations of antimicrobial resistance based on two complementary tools with default parameters: (i) abricate v1.0.1 (https://github.com/tseemann/abricate using the Virulence Factor Database (42) (accessed 2020-04-19) and MEGARes v2.0 (43) as references; and (ii) DeepARG v1.0 (44).

Mobile genetic elements were identified by annotating recombinases using 68 high-accuracy profile HMM models and reconciling these results using pangenome information as described in (25). This yielded ∼33 million MGE recombinases across the entire database of which the ones belonging to representative genomes were subsequently used to annotate MGE types namely transposable elements, integrons, phages and conjugative elements including plasmids within the representative set.

Biosynthetic gene cluster (BGC) prediction was performed with GECCO v0.9.5 (45), using features from Pfam v35.0 (46).

### Habitat information

Consistent habitat annotations are becoming more and more important for genomics analyses (4). Thus, proGenomes3 provides annotations of genomes and specI species clusters to habitats. For proGenomes3, we updated the habitat annotation process which now includes annotation based on both the PATRIC database (47) and the Microbe Atlas Project (MAP) (17).

**Figure 2.**
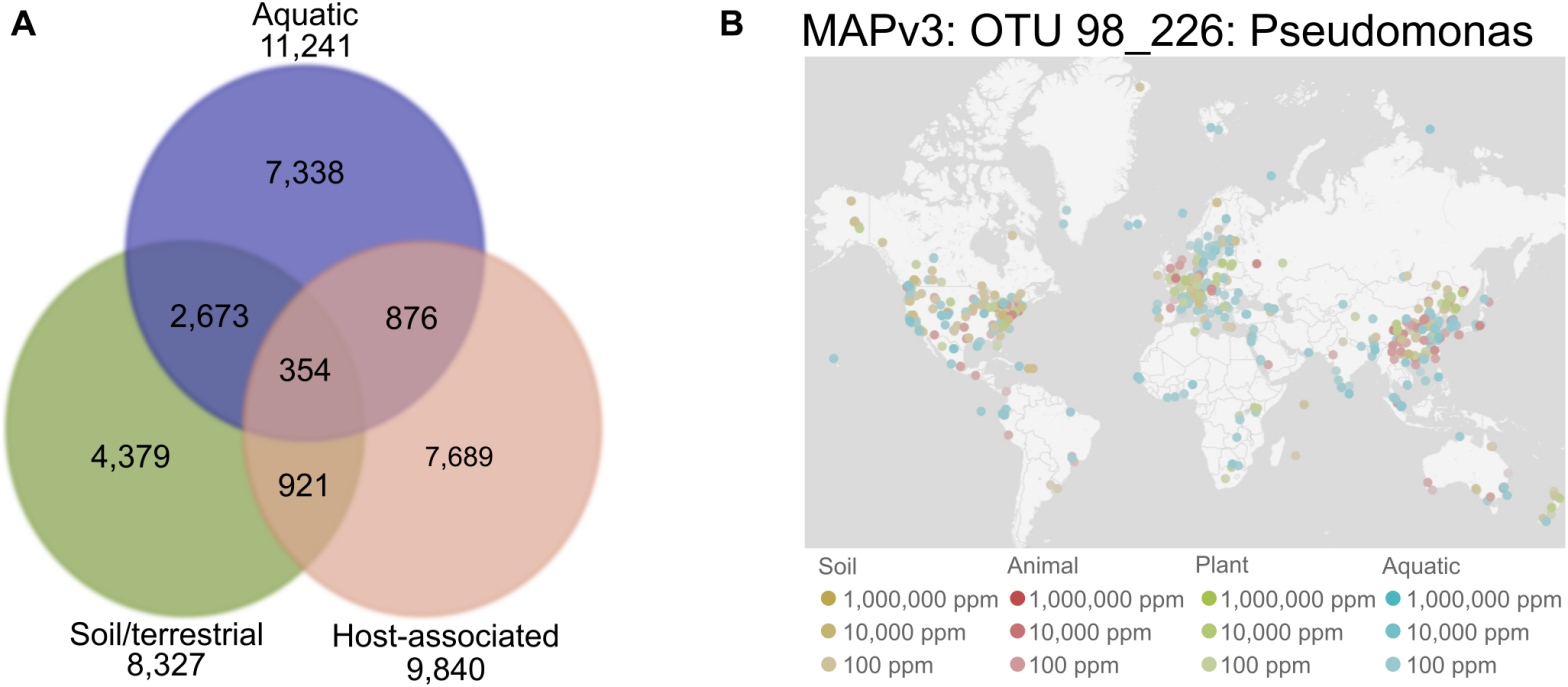
Habitat annotations. (**A**) Venn diagram of specI clusters annotated to different high-level habitat categories. (**B**) Global distribution of one MAP OTU linked to a specI cluster in proGenomes.

For habitat annotations based on the PATRIC database, information regarding the isolation source was parsed from the PATRIC database version 3.6.12 (accessed on 29 August 2022). PATRIC habitat annotations are available for 25 314 out of the 41 171 specI clusters (187 808/907 388 genomes) with three main categories (soil-associated, aquatic, host-associated, Figure 2A) and several additional categories (mud/sediment, freshwater, disease-associated and food-associated). In more detail, we downloaded the PATRIC metadata including all metadata fields. The PATRIC habitat metadata was curated by finding key-words that allow to place an isolate into one of the habitat categories (‘soil’, ‘aquatic’, ‘host-associated’) in any of the columns ‘habitat’, ‘isolation_source’, ‘disease’ in the downloaded file from the PATRIC database.

For Microbe Atlas Project (MAP) annotations, we extracted 16S rRNA genes from the proGenomes3 genomes and matched them to the set of MAP OTUs clustered at 98% ID. When multiple 16S rRNA genes were found, the longest version was selected. 636 792 (84.5%) of the 753 909 16S rRNA (longer than 600 bp) sequences identified in proGenomes3 confidently mapped to 16 366 MAP OTUs. The mapped 16S sequences were furthermore analyzed to create links between specI clusters and 98% MAP OTUs. A majority rule was employed to identify the best match for each specI cluster. A link was only generated if at least 80% of the 16S sequences within one specI cluster were mapped to the same 98% MAP OTU. This led to a reliable assignment of 19 902 specI clusters to 9511 MAP OTUs with habitat information. In proGenomes3, we link to the MAP website which also enables the visualization of the world-wide distribution of MAP OTUs (Figure 2B).

As before we compiled sets of representative genomes for different habitats which can be downloaded directly from the proGenomes website.

### Links to outside databases

Dedicated databases often provide very detailed information which is not mirrored in proGenomes3. To accommodate easier access to this information, we added additional links to outside databases such as NCBI Genome (48), BacDive (49), GTDB (12) and MAP (17).

### Database design

The core of proGenomes is a relational database system powered by PostgreSQL, which stores all relevant information on the included genomes and their features which are available through the web user interface. Due to its size (close to 8 Tb), the sequence information (genomes, gene and protein sequences) is stored in custom indexed FASTA flatfiles. This allows the retrieval and download of user requested individual sequences with acceptable response times.

### Website

proGenomes3 can be accessed via its dedicated website (http://progenomes.embl.de). The genomes of taxonomic groups as well as specI clusters can be accessed easily via a search function. For each genome, we provide the information stored within proGenomes3 as well as direct links to external database entries.

As in previous versions, user-supplied genomes can be taxonomically annotated using the same placement algorithm as described previously for proGenomes2.

### Future outlook

We are constantly improving proGenomes and will continue to do so in the future. Our goal is to provide even richer annotation sets as well as datasets that can be used for data science applications for microbial genomes. One major focus will be on the ever-growing number of MAGs has motivated plans for their inclusion in future proGenomes releases.

## DISCUSSION

proGenomes3 provides nearly one million high-quality genomes with consistent taxonomic, functional, and habitat annotations. These data can be accessed via a dedicated website that also provides additional information such as links to other relevant databases or by direct download of sets of representative genomes (general and habitat specific). proGenomes continues to facilitate comparative studies addressing questions from evolution, population genetics, functional genomics and many other research fields for researchers at all levels of experience in genomics. Previous versions have been used to establish important resources such as eggNOG (20), mOTUs (50,51) and the Global Microbial Gene Catalog (52), while being used in research projects that led to impactful discoveries (4,53–55)

Hence, we expect proGenomes3 to be a valuable resource for many upcoming studies ranging from those focusing on one or a few organisms to those analyzing large-scale evolutionary patterns or complex microbial communities.

## DATA AVAILABILITY

No new data were generated or analysed in support of this research. proGenomes3 is available at https://progenomes.embl.de/.
